# Endoscopic Microvascular Decompression for Hemifacial Spasm: A Technical Case Report Demonstrating the Benefits of the Angled Endoscope and Intraoperative Neuromonitoring

**DOI:** 10.7759/cureus.16586

**Published:** 2021-07-23

**Authors:** Rachel Blue, Susanna Howard, Michael Spadola, Svetlana Kvint, John Y.K. Lee

**Affiliations:** 1 Neurosurgery, Hospital of the University of Pennsylvania, Philadelphia, USA

**Keywords:** endoscope, microvascular decompression, hemifacial spasm, minimally invasive, lateral spread response, neuromonitoring

## Abstract

A 57-year-old female with eight years of hemifacial spasm (HFS) underwent endoscopic microvascular decompression (MVD) of the facial nerve. Baseline stimulation of the zygomatic branch of the facial nerve activated at 1.2 mA. Lateral spread response (LSR) to the buccal and mandibular branches was observed at 2.2 mA. A straight endoscope was used to enter the cerebellopontine angle, allowing for visualization of the vestibulocochlear and facial nerve. Neurovascular compression was not clearly identified. A 30-degree endoscope was directed medially/inferiorly and compression at the root entry zone was identified and decompressed. Subsequent LSR to the buccal/mandibular branches was seen at 3.2 mA/3.6 mA, respectively. Additional vascular compression was suspected given persistent LSR. The 30-degree endoscope was directed laterally. Compression was seen at the porus acustics and decompressed. Subsequent LSR to the buccal/mandibular branches was not observed until 9.8 mA, indicating good decompression. The patient tolerated the procedure well with complete resolution of her symptoms and remains spasm-free as of three months post-procedure without a hearing deficit. The 30-degree endoscope enabled visualization of pathology that was not easily seen at 0-degree. Additionally, LSR indicated persistent nerve compression following root entry zone decompression. Subsequent distal decompression resulted in greater LSR reduction. This case report suggests that MVD for HFS may yield better results with both proximal and distal decompression of the seventh nerve, and this type of decompression can benefit from endoscopic visualization.

## Introduction

Hemifacial spasm (HFS) is a condition of unilateral involuntary, spasmodic contractions of the muscles innervated by the facial nerve. It typically begins with the orbicularis oculi muscle with eyelid closure/eyebrow elevation and spreads to involve the frontalis, platysma, and orbicularis oris muscles [1-2]. The symptoms of HFS can be progressive and associated with psychosocial distress, vision disturbances, sleep difficulties, and a lower quality of life [3]. Classically, the etiology of HFS is attributed to compression of the facial nerve at the root exit zone at the brain stem by ectatic or aberrant arteries of the posterior cerebral and/or cerebellar circulation [4].

In moderate to severe cases of HFS, medical management is often ineffective and microvascular decompression (MVD) is the preferred treatment modality, either by an endoscopic or a microscopic approach. With an endoscopic approach, angled lenses can be used to better identify pathological neurovascular compression. Intraoperatively, neurovascular decompression can be assessed visually and by way of monitoring lateral spread response (LSR). This case report highlights how the endoscopic MVD approach and LSR can be used to optimize the neurosurgical management of patients with HFS.

## Case presentation

**Figure 1:**
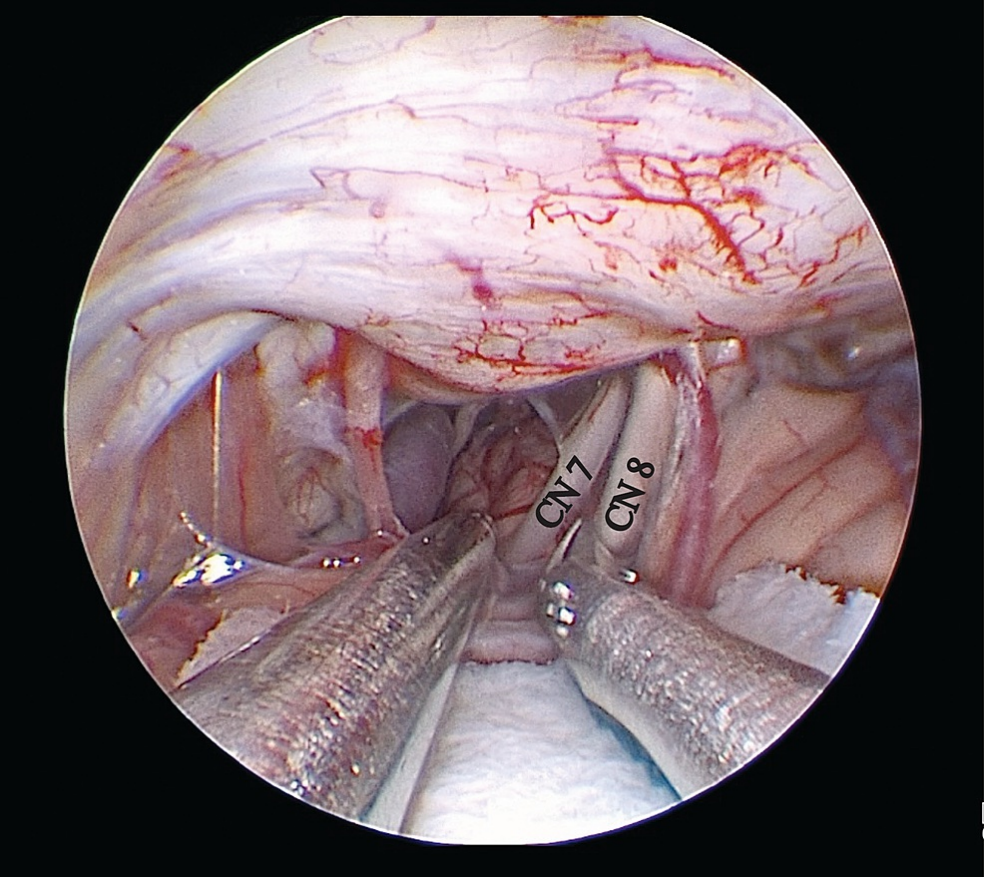
Initial approach with 0-degree endoscope -- no neurovascular compression identified. CN, cranial nerve

A 57-year-old female with no significant past medical history presented with eight years of left-sided classic HFS. The patient attempted medical management, including multiple rounds of botox, with some benefit, however, she continued to experience bothersome twitching in between injections. After a discussion of the risks and benefits of the treatment options, she decided to proceed with endoscopic MVD of the facial nerve. The patient was taken to the operating room and neuromonitoring needles were placed. A keyhole retrosigmoid craniectomy was drilled in a standard fashion. Baseline neuromonitoring was achieved through stimulation of the zygomatic branch of the facial nerve, which activated at 1.2 mA. LSR of the buccal and mandibular branches was activated at 2.2 mA stimulation. A straight 2.7 mm outer diameter endoscope was used to enter the cerebellopontine angle. Cerebrospinal fluid (CSF) was drained for relaxation and the lower cranial nerves were identified. The arachnoid was dissected allowing for visualization of cranial nerves seven and eight. Neurovascular compression of the facial nerve was suspected but not identified (Figure 1).

**Figure 2:**
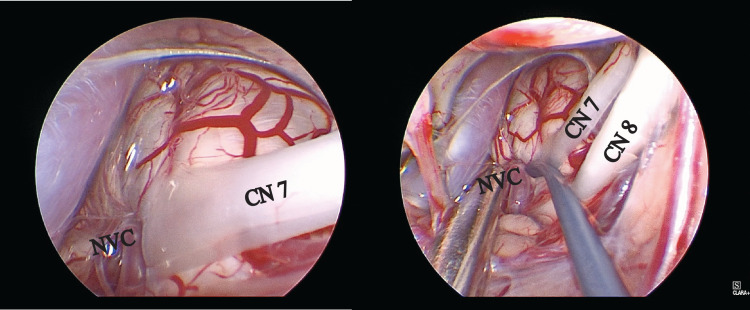
Medial and inferiorly directed 30-degree endoscope with identification of neurovascular compression at the root entry zone of the facial nerve. CN, cranial nerve; NVC, neurovascular compression

A 30-degree endoscope was directed medially and inferiorly and neurovascular compression at the root entry zone of the facial nerve was identified (Figure 2).

**Figure 3:**
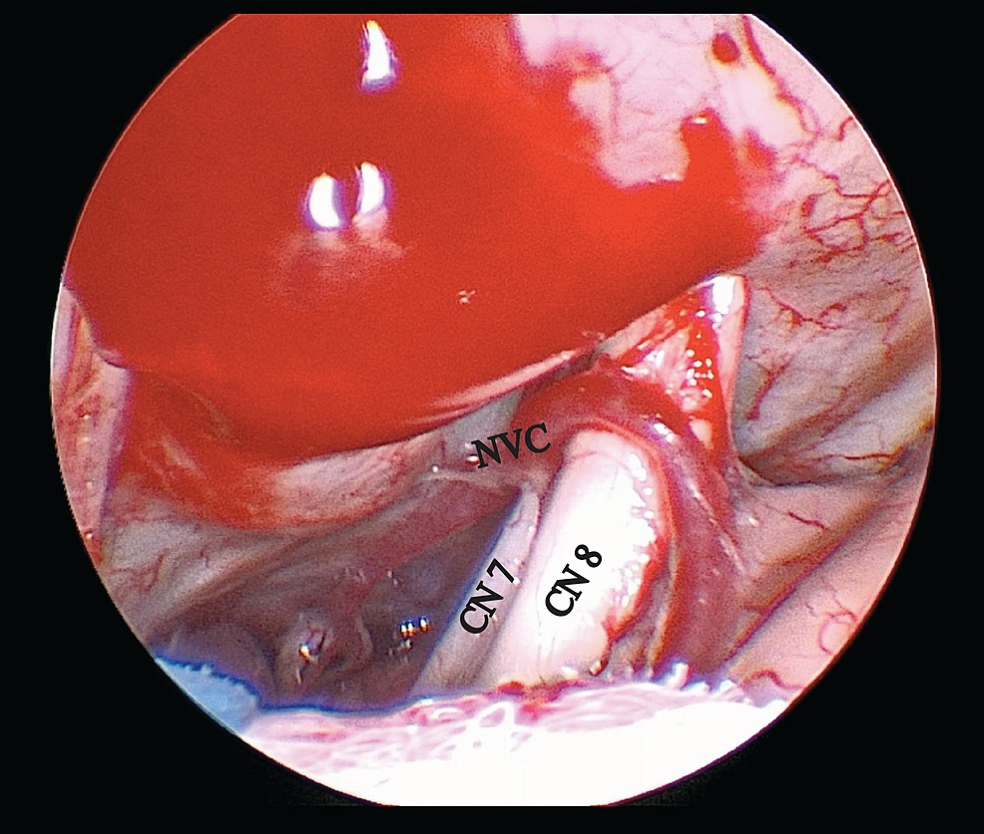
Lateral and superiorly directed 30-degree endoscope with identification of a second area of neurovascular compression at the distal portion of the facial nerve. CN, cranial nerve; NVC, neurovascular compression

This was then decompressed with three pieces of shredded polytetrafluorethylene (PTFE). Following decompression, the LSR to the buccal nerve was present at 3.2 mA stimulation, and to the mandibular branch at 3.6 mA simulation. While this was an improvement from baseline, significant LSR was still present. As such, the angled endoscope was directed lateral (distally along the seventh nerve) to assess for additional areas of compression (Figure 3). A second distinct area of neurovascular compression was seen at the porus acusticus and was decompressed with a single piece of PTFE (Video 1).

**Video 1:** Endoscopic microvascular decompression for HFS: angled endoscope. HFS, hemifacial spasm

Following decompression, the LSR of the buccal and mandibular branches were not activated until 9.8 mA stimulation intensity, indicating good decompression. The patient tolerated the procedure well without complications and had complete resolution of her symptoms. 

## Discussion

Historically, MVD has been performed using the operating microscope and a retrosigmoid approach to gain access to the facial nerve [5-6]. In a review of 22 studies including 5700 patients, MVD resulted in complete resolution of symptoms in 91% of patients with HFS with low rates of symptom recurrence [3]. While the success rates are high and complications rare with microscopic MVD, illumination and visualization are restricted to a direct line of site, which can necessitate a larger craniotomy and increased retraction on surrounding structures [7-9]. Additionally, this lends to the potential for missed pathology that is not directly visualized with the microscope. These limitations can be overcome during the MVD procedure by using an endoscope which provides enhanced visualization and the ability to see around corners to more accurately assess the completeness of decompression [7, 9-12]. The endoscope has the ability to be advanced in the surgical corridor, expanding the visualization of pathology. Further, angled lenses can be used to see around corners if necessary. In a series of 60 patients, the site of vessel-nerve compression was identified in 93% of cases when the endoscope was used. In this same group of patients, the site of compression could only be identified in 28% of cases when the microscope was used [9].

This case report demonstrates the use of the 30-degree endoscope enabling visualization of pathology that otherwise was not seen at 0-degree, both at the root entry zone and at the distal portion of the nerve. This visualization allowed for complete nerve decompression. While the endoscope offers a clear advantage with an improved line of site, smaller craniotomies, and less cerebellar retraction, it does require nuanced surgical technique with regard to safe advancement into the surgical corridor, instrument working room around the endoscope, and visualization in 2D rather than 3D, all of which has a learning curve for surgeons without endoscopic experience [11].

Another technical advance in MVD for HFS involves intraoperative neurophysiological testing of facial nerve function. When a branch of the facial nerve is electrically stimulated during surgery, the pattern of induced facial muscle responses is often abnormal in patients with HFS. Muscles that are not directly innervated by the stimulated facial nerve branch contract as a result of the abnormal spread of electrical activation to other nerve branches. This pathological LSR can be measured to assess the efficacy of the facial nerve decompression intervention [2, 11, 13-16]. When measured intraoperatively, the LSR can be greatly reduced or disappear when the compressing vessel is repositioned away from the facial nerve [14, 17-18]. There is evidence that reduction or elimination of the LSR after MVD is associated with the resolution of symptoms and low rates of recurrence [13, 19]. A meta-analysis of 855 patients who underwent MVD for HFS showed that the failure rate among patients in which LSR did not resolve was 39% compared to 9.5% among patients in which LSR did resolve [20].

In this case, the use of intraoperative neuromonitoring of LSR guided the surgeon to search for an additional area of pathology that was distinct from the classical compression noted at the root entry zone. Interestingly, the second distal decompression had a greater response based on LSR, which is contradictory to the traditional belief that the pathophysiological compression of HFS occurs at the root entry zone of the facial nerve. This case report suggests that vascular compression for HFS may yield better results with both proximal and distal decompression of the seventh nerve, and this type of decompression can benefit from endoscopic visualization.

## Conclusions

The addition of the endoscope in skull base surgery has allowed for greater visualization, smaller craniotomies, and lesser retraction on adjacent tissues. This case demonstrates the use of the angled endoscope to identify pathology that would have otherwise been missed. Additionally, the use of intraoperative neuromonitoring of LSR enabled real-time assessment of neurovascular decompression and suggests that pathological compression may not be limited to the root entry zone of the facial nerve.
